# Prevention for oneself or others? Psychological and social factors that explain social distancing during the COVID-19 pandemic

**DOI:** 10.1177/1359105320980793

**Published:** 2020-12-10

**Authors:** Natalie Christner, Regina M Sticker, Lena Söldner, Maria Mammen, Markus Paulus

**Affiliations:** Ludwig-Maximilians-Universität München, Germany

**Keywords:** Covid-19, empathy, moral identity, norms, social distancing

## Abstract

Identifying the underlying psychological and social factors of social distancing is crucial to foster preventive behavior during a pandemic effectively. We investigated the relative contribution of self-focused factors (fear of infection, fear of punishment) and other-focused factors (moral judgment, moral identity, empathy for unspecific others, empathy for loved ones) in an online study in Germany (*N* = 246) while COVID-19 was climaxing. Importantly, other-oriented factors were related to social distancing behavior beyond self-oriented factors. Moral judgment and empathy for loved ones remained the dominant factors while controlling for all aspects. These findings underline the relevance of interpersonal considerations when engaging in preventive behavior.

The pandemic caused by the SARS-CoV-2 virus is a worldwide health threat and causes major changes in everyday life (Blom et al., 2020; Windsteiger et al., 2020). Until there is a cure or vaccine for the disease, the most effective way to prevent a collapse of the healthcare system, which would lead to many deaths, appears to be social distancing (Greenstone and Nigam, 2020). Social distancing means keeping physical distance from others, including family and loved ones. This seemingly easy method seems rational in the current situation. Yet, the required behavior is exactly the opposite of human tendencies when confronted with a crisis. When being threatened, humans naturally tend to seek social contact and proximity to others (Dezecache et al., 2020; Mikulincer and Shaver, 2003). Therefore, it is important to investigate which psychological and social factors raise the acceptance of social distancing and thus facilitate handling a global health crisis.

The World Health Organization (2020) recommends social distancing to slow the spread of the SARS-CoV-2 virus. Most governments implemented methods to reduce physical contact between their citizens, which largely apply up until today, such as cancelling public events. Many countries forbade gatherings of two or more people from different households (Robert Koch Institute, 2020; World Health Organization, 2020). For example, violating these rules in Germany could have led to a fee of 150€ or more (e.g. Bayerische Staatskanzlei, 2020). Most citizens have adhered to the restrictions from early on, but there was considerable variance in acceptance (Betsch et al., 2020a).

By distancing oneself from potentially infectious others, social distancing lowers one’s own risk of getting infected. Moreover, in regions that fine deviation from social distancing, adhering to social distancing is preventing oneself from punishment. In that sense, social distancing can be regarded as selfish behavior, with fear of getting infected or fear of punishment being a driving force. Indeed, several researchers identified fear of an infection as a key predictor of social distancing (Harper et al., 2020; Lin et al., 2018). Considering such self-focused motives during a pandemic is thus important to effectively target citizens’ motivation to adhere to preventive behaviors.

Besides self-focused reasons, other-focused concerns might impact social distancing. Since people can spread the virus unknowingly, even (seemingly) healthy individuals should keep distance from others to lower the risk of transmitting the virus (Koo et al., 2020; Wilder-Smith and Freedman, 2020). In that sense, social distancing can be seen as a form of other-oriented or prosocial behavior that aims at the well-being of others above the self-focused motive. Recent studies highlighted the prosocial aspect of preventive behavior. Framing the benefit of preventive behavior in public/prosocial rather than personal terms (e.g. “avoid spreading” vs “avoid getting COVID-19”; Jordan et al., 2020) and framing the risk with regard to vulnerable people rather than the transmission rate in general (Lunn et al., 2020) has shown to increase preventive intentions. In addition, a study by Francis and McNabb (2020) supports the idea that everyday behavior such as hygiene or social distancing behavior received moral value during the COVID-19 pandemic. Moralizing these behaviors was positively related to actually following the behavior (Francis and McNabb, 2020). Furthermore, empathy seems to motivate distancing behavior (Pfattheicher et al., 2020). In times of a pandemic, social distancing thus can be regarded as prosocial behavior.

These findings pose the question about which psychological mechanisms ultimately drive social distancing, and, importantly, about their relative contribution. The question whether self-focused motives or concern for other’s welfare primarily drive behavior is highly interesting, as it relates to a perennial question in human history. Are people rather self-focused and egoistic, or motivated by moral evaluations and other-related concerns (Aristotle, trans. 2009; Plato, trans. 2008). Indeed, the relation between psychological egoism and altruism remains topic of a vivid debate in modern and contemporary philosophy (Bentham, 1789/2007; Nagel, 1970; Schopenhauer, 1840/1995). With this study, we aim to examine the relative contribution of self-focused and other-focused behavioral motives in the context of a global pandemic.

Psychological and social factors promote prosocial behavior, including cognitive factors such as moral judgment and moral identity, and rather affective factors such as empathy. Moral judgments inform about how people evaluate behavior and they also relate to the corresponding behavior (Killen and Dahl, 2018; Turiel, 2015). In particular, considering a behavior as moral instead of conventional or personal preference can explain behavioral tendencies (Francis and McNabb, 2020; Rhee et al., 2019; Smetana, 1982). Already preschoolers evaluate transgressions affecting human welfare particularly severely (Smetana, 1981) and develop a normative concern for the well-being of others (Paulus et al., 2020). But initially neutral behaviors of personal preference can also gain moral significance anytime, that is, become moralized (Rhee et al., 2019). These theories and findings are relevant for the current study, because the intensity of social distancing behavior, such as avoiding to invite friends, likely was a question of personal preference before the pandemic. However, with increasing prevalence of the virus, these behaviors became a topic of human welfare. Moralization is therefore important to consider when trying to explain behavior. That means, the more someone considers a behavior to be morally relevant, the more likely he or she might act accordingly.

While people might come to a similar moral judgment about a certain behavior, the degree to which being a moral person is central to someone, the moral identity, differs between individuals (Hardy and Carlo, 2011; Lapsley and Narvaez, 2004). Moral identity is suggested to bridge a gap between moral judgment and behavior (Blasi, 1983). That means, particularly when morality is central to one’s self, moral judgments (e.g. “Sharing is good”) are translated to actual behavior (e.g. donating money). Particularly the internalization of moral identity, that is, the degree to which being a moral person is considered a personal striving (Aquino and Reed, 2002), appears to relate to moral behavior (Boegershausen et al., 2015; Hertz and Krettenauer, 2016). For example, people with a strong internalization seem to have a wider “circle of moral regard.” Based on their relevance for other-oriented behavior, moral identity and moral judgment are two factors that we investigated in the context of social distancing.

In addition, previous literature on prosociality highlights empathic concern or sympathy as one driving factor of prosocial behavior (Batson et al., 1981; Davis, 1983; for review see Eisenberg et al., 2010). According to Batson (2011), empathy describes the concern for the well-being of others and leads to altruistic motivation, meaning the motivation to increase other’s welfare. We will use this definition for the current study. Pfattheicher (2020) assessed the relation between empathy with those vulnerable to COVID-19 and social distancing during this pandemic. They found that the more empathy participants reported, the more they reported to practice social distancing, highlighting the role of empathy for preventive behavior.

Recent theoretical work highlights that empathy should also be considered a relational phenomenon (Betzler, 2019). Empathy can result in valuable relationships and particularly close relationships call for empathy. We thus hypothesized that it is particularly empathy for close others that affects the tendency to keep physical distance from others during the pandemic. Differentiating between empathy for unspecific vulnerable others and empathy for loved ones (e.g. family and friends) allows to pinpoint the underlying factors of social distancing in detail.

## The current study

The current study aimed to investigate the relative contribution of psychological and social factors that are associated with social distancing during the COVID-19 pandemic. We examined the relative contribution of self-focused factors—aiming at maximizing one’s own well-being—and other-oriented factors—aiming at moral considerations and the well-being of others. Our central research question was: Are other-focused factors related to preventive behavior in a global pandemic beyond self-focused factors? As self-focused factors, we considered fear of infection and fear of punishment. As other-oriented factors, we considered moral judgment, moral identity, empathy with unspecified vulnerable others, and empathy with loved ones. We expected other-oriented factors to have a greater contribution to social distancing than self-oriented factors, because social distancing might become a moral act during a pandemic. Within the other-oriented factors, we examined the relative contribution of cognitive (i.e. moral judgment and identity) and affective aspects (i.e. empathy), as theories highlight the relevance of both for other-oriented behavior (Batson, 2011; Lapsley and Narvaez, 2004; Turiel, 2015).

Next to this general question, we considered one specific interaction between two moral factors. Following the theory on moral identity (Blasi, 1983), we expected moral identity to moderate the link between moral judgment and behavior. If being a moral person is important to oneself, the urge to stay self-consistent might lead to behavior that corresponds one’s moral judgment. We thus expected that particularly if being a moral person is central to a person’s identity, considering social distancing as moral should lead to social distancing.

We addressed our research question in an online study in a German sample in mid-April 2020, as the infection rate was climaxing. During the time of data acquisition and up until now, in Germany, social distancing was and still is enforced through fines.

## Method

### Participants

The final sample comprised 246 participants (176 female; *M*_age_ = 37.1, *SD*_age_ = 14.4), who lived in Germany at the time of data collection. Half the participants (50%) were currently working from home, 11% worked with contact to patients or customers, 9% could not execute their job due to the pandemic, and 16% did not work. Two additional participants completed the questionnaire but did not pass an attention check question (see below). Participants were recruited via online postings (websites, social media) and by word of mouth. An a-priori power analysis revealed a minimal sample size of 163 to detect an effect of *f*^2^ = .08 with α = 0.05 and power of 0.95. We expected this effect size for the relation of moral identity with social distancing beyond empathy, moral reasoning, age, and gender based on the findings by Hardy (2006) on prosocial behavior. The local ethics committee approved the study. Participants provided informed consent online.

### Procedure and design

Participants completed an online questionnaire via the platform Qualtrics. Participation took around 10 minutes. Participants had the chance to win one of three book vouchers.

### Measures

Our dependent variable was social distancing behavior. As independent variables, we assessed other-oriented factors (moral judgment, moral identity, empathy in general, empathy for loved ones) and self-oriented factors (fear of infection, fear of punishment). A full list of the items is available in the Supplemental Material. After about half the questions, we included an attention check question. We excluded participants who failed to answer the check question correctly. All scales were created by computing the mean across items.

#### Social distancing

Participants reported their social distancing behavior on six items via self-report (e.g. “I minimize physical contact to others (so-called “Social Distancing”). Some items were adapted from Pfattheicher et al. (2020). We expanded the social distancing questionnaire by five distractor items. Participants responded on a 7-point Likert scale (*not true at all*—*completely true*) with higher values indicating more regular practice of social distancing.

#### Moral judgment

We assessed whether participants judged the act of social distancing in moral terms. In four items, we asked participants to indicate how morally relevant they considered social distancing to be. One of these items addressed morality in agreement with the notion that moral norms are universal and independent of authorities or laws (Turiel, 2015; e.g. “Even if there were no state regulations about ‘Social Distancing’, ‘Social Distancing’ would be morally required in the current situation.”).

#### Moral identity

To assess moral identity, we employed the Self-Importance of Moral Identity questionnaire (SMI-Q) by Aquino and Reed (2002). This measure includes 10 items consisting of the subscales *Internalization* and *Symbolization* (5 items each). All items were reported on a 7-point Likert scale (*strongly disagree*—*strongly agree*) with higher values indicating a stronger moral identity. In line with previous research (Aquino et al., 2009; DeCelles et al., 2012), we considered the *Internalization* mean as the moral identity score (moral identity-I), because this subscale is considered most relevant for behavior.

#### Empathy (general)

We used three items from Pfattheicher et al. (2020) to assess empathy for unspecific vulnerable others (e.g. “I feel compassion for those most vulnerable to COVID-19.”).

#### Empathy (loved ones)

We implemented three items to assess empathy for loved ones (e.g. “I am very concerned about family members or friends who are especially vulnerable to COVID-19.”).

#### Fear of infection

We assessed how worried people were about infecting themselves with COVID-19 by adapting items from the Whiteley-Index, an instrument for assessing hypochondriasis (Hinz et al., 2003). In four items, we asked participants to report their fear of infection (e.g. “I often worry that I might contract COVID-19.”).

#### Fear of punishment

During the survey period, disregarding the state-ordered social distancing regulations could lead to a fine. We included two items to assess fear of punishment (e.g. “I worry that I might get fined if I do not adhere to the state-ordered lockdown rules and ‘Social Distancing’”).

For moral judgment, the empathy measures, fear of infection, and fear of punishment, answers were given on a 5-point Likert Scale (*I disagree*—*I strongly agree*).

#### Demographic variables

Because some people are particularly threatened by Covid-19, we asked participants to indicate whether they belonged to an at-risk group (yes/no/don’t know). Additionally, we assessed participant’s age (in years), gender (0 = female; 1 = male), and highest education degree. Furthermore, we asked participants about their current work situation and in which federal state of Germany they lived.

### Data sharing statement

All questionnaire data and the analysis script are available on https://osf.io/sxaq5/. The study was not preregistered.

## Results

Table 1 presents descriptive statistics of the key variables and scale reliabilities.

**Table 1.** Cronbach’s α as a measure of internal consistency, means, and standard deviations for key variables.

Variable	α	*M*	*SD*	Scale
Social distancing	.65	6.15	0.84	1–7
Moral identity-I	.75	5.84	0.79	1–7
Moral judgment	.86	4.16	0.78	1–5
Empathy (general)	.89	4.07	0.87	1–5
Empathy (loved ones)	.80	3.73	0.98	1–5
Fear of infection	.83	2.65	0.98	1–5
Fear of punishment	.75	2.75	1.20	1–5

Scale indicates the range of possible values for each item of a scale. Lower values on each scale reflect a lower degree, and higher values reflect a higher degree of the respective variable.

### Demographic variables

Most participants (79%) reported that they were not part of the at-risk group for Covid-19. Participants at-risk or who didn’t know about their risk status reported more fear of becoming infected (*M* = 3.02, *SD* = 0.99) compared to participants not at risk (*M* = 2.56, *SD* = 0.96), *t*(244) = 3.07, *p* = 0.002, *d* = .48. The two groups did not differ regarding social distancing, *t*(244) = 0.36, *p* = 0.716, or any other key variable, *p*s > 0.180. Most participants reported holding a university degree (74%). Participants with a university degree reported slightly more fear of getting infected (*M* = 2.73, *SD* = 0.98) compared to those without a university degree (*M* = 2.44, *SD* = 0.98), *t*(241) = 1.98, *p* = 0.049, *d* = .29. The two groups did not differ regarding social distancing, *t*(241) = 0.005, *p* = 0.996, or any other key variable, *p*s > 0.279. We will address effects of gender and age in the following correlation and regression analyses.

### Main variables

Table 2 presents zero-order correlations between the main variables for descriptive purpose. Social distancing correlated positively with moral identity, moral judgment, empathy in general, empathy for loved ones, and fear of infection, but it did not correlate with fear of punishment. To investigate the relative contribution of all factors for social distancing, we computed a hierarchical linear regression with mean-centered variables. Some predictors correlated highly, but we kept them distinct for conceptual reasons. Moreover, the Variance Inflation Factor was below 2 for all predictors in all models, indicating no problem of collinearity (Fahrmeier et al., 2013). In step 1, we entered the control variables age (in years) and gender as well as the self-focused factors fear of infection and fear of punishment. In step 2, we added moral identity, moral judgment, empathy in general, and empathy for loved ones to examine whether these other-focused factors are related to social distancing beyond self-focused factors. In step 3, we added the interaction term of moral identity and moral judgment to investigate the moderation.

**Table 2.** Zero-order correlation matrix with two-tailed Pearson correlations.

	1	2	3	4	5	6	7	8
2	0.16*	-						
3	0.50***	0.25***	-					
4	0.22***	0.29***	0.37***	-				
5	0.34***	0.23***	0.36***	0.63***	-			
6	0.20**	0.21***	0.33***	0.44***	0.51***	-		
7	−0.02	0.01	−0.15*	0.07	0.14*	0.05		
8	−0.13*	−0.06	0.01	0.04	0.00	0.05	−0.24***	
9	−0.12^+^	−0.16*	0.00	−0.17**	−0.17**	−0.01	−0.17**	0.10

1: Social distancing; 2: Moral identity-I; 3: Moral judgment; 4: Empathy (general); 5: Empathy (loved ones); 6: Fear of infection; 7: Fear of punishment; 8: Age; 9: Gender [0 = female; 1 = male].

* *p* < 0.05. **p < 0.01. ***p < 0.001. ^+^*p* < 0.10.

Table 3 shows the regression results. Fear of infection was positively related to social distancing at step 1. This effect, however, vanished when adding all other predictors at step 2. Even though moral identity correlated with social distancing (see Table 2), the relation became non-significant when adding the other moral factors at step 2. Moral judgment, that means, whether social distancing is considered as morally relevant, and empathy for loved ones thus remained the dominant predictors of social distancing.

**Table 3.** Hierarchical linear regressions on social distancing behavior with standardized regression coefficient, *p*-value, and 95% confidence interval for each predictor.

	Social distancing								
	Step 1			Step 2			Step 3		
	*β*	*p*	95% CI	*β*	*p*	95% CI	*β*	*p*	95% CI
Age	**−.15**	0.022	[−.02, –.00]	**−.13**	0.027	[−.01, –.00]	**−.13**	0.024	[−.01, –.00]
Gender	−.12	0.069	[−.44, .02]	−.09	0.130	[−.37, .05]	−.07	0.187	[−.34, .07]
Fear of infection	**.21**	0.001	[.07, .28]	−.02	0.714	[−.13, .09]	−.01	0.916	[−.11, .10]
Fear of punish.	−.09	0.188	[−.15, .03]	−.02	0.728	[−.09, .07]	−.01	0.852	[−.09, .07]
Moral identity-I				.00	0.934	[−.12, .13]	−.00	0.958	[−.12, .12]
Moral judgment				**.46**	0.000	[.36, .62]	**.45**	0.000	[.35, .61]
Empathy (general)				−.11	0.149	[−.24, .04]	−.10	0.158	[−.23, .04]
Empathy (loved)				**.24**	0.002	[.08, .33]	**.23**	0.003	[.07, .32]
Moral judgment × moral identity-I							**−.14**	0.011	[−.35, −.05]
Δ*R*^2^, *p*	.08	0.001		.23	0.000		.02	0.011	
*R*^2^, *p*	.08	0.001		.31	0.000		.33	0.000	

Coefficients in bold, if *p* < 0.05.

**Figure 1.**
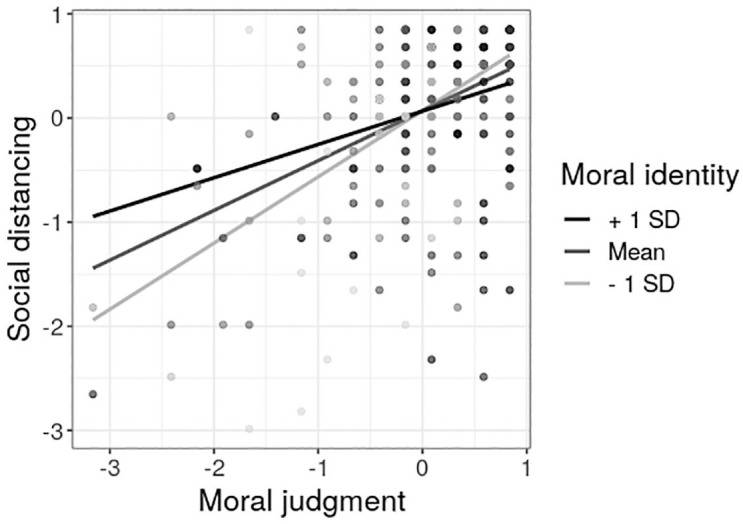
Interaction of moral judgment and moral identity on social distancing behavior on mean-centered scores (zero on *x*- and *y*-axis reflects sample mean of the respective variable). Slopes are depicted for three levels of moral identity: low (−1 *SD*), medium (mean), high (+1 *SD*).

Step 3 revealed a significant interaction of moral identity and moral judgment. To follow up on the interaction, we computed simple slope analyses for a low, medium, and high level of moral identity (−1 *SD*, mean, +1 *SD*; see Figure 1). All three slopes differed significantly from zero (low moral identity: *b* = .64, *p* < 0.001, 95% CI [.47, .81]; medium moral identity: *b* = .48, *p* < 0.001, 95% CI [.35, .61]; high moral identity: *b* = .32, *p* < 0.001, 95% CI [.13, .51]). The slope was most positive for a low level of moral identity. That means, the lower participant’s moral identity, the more considering social distancing as morally relevant increased social distancing. The overall pattern of results does not change if risk-status and educational degree are added to the model as control variables at step 1.

## Discussion

Social distancing is deemed an effective method to slow the infection rate during a pandemic. Identifying the main psychological and social factors that motivate people to follow social distancing regulations throughout a health crisis is therefore highly important. The current study examined the relative contribution of other-oriented factors (moral judgment, moral identity, empathy in general and for loved ones) and self-oriented factors (fear of infection, fear of punishment) to social distancing during the COVID-19 pandemic. All other-oriented factors and fear of infection were positively related to social distancing. However, considering all factors simultaneously identified moral judgment and empathy for loved ones as the main factors related to social distancing. These findings suggest that even in times when everyone’s health is threatened, mainly the moral relevance of the situation and concern for others guide preventive behavior.

Other-oriented factors outweigh self-oriented factors in being associated with social distancing. This main finding underlines the relevance of interpersonal considerations and moral reflection even in face of a severe crisis. Moral judgment, conceptualized as considering social distancing as morally relevant, was the most important factor, thereby highlighting the behavioral relevance of moral judgment (Killen and Dahl, 2018). Additionally, the finding that social distancing behavior gained moral significance, which in turn was linked to behavior, fits to moralization research (Francis and McNabb, 2020; Rhee et al., 2019). The findings thus highlight the importance of considering social distancing as moral behavior.

Next to moral judgment, empathy for loved ones remained a dominant factor for social distancing. Importantly, the findings complement the results by Pfattheicher et al. (2020), who demonstrated that empathy for unknown others leads to social distancing. In our study, empathy in general was not most relevant for social distancing, as the relation vanished when controlling for other factors. Particularly empathy for loved ones was associated with behavior. Hence, the extent to which this affective factor is indeed other-oriented can be discussed. Close relationships are characterized by reciprocity and theories suggest that empathy helps to deepen close relationships (Betzler, 2019; Laursen and Hartup, 2002). Moreover, current theories suggest that close relationships come with a normative obligation to be partial (Betzler, 2014; Scheffler, 2010). Empathy for close others might thus benefit oneself in the long run. But since empathy reflects by definition a concern for others, we consider also empathy for loved ones as a motivation that focuses on the well-being of others. Moreover, self-focused factors in the current study were egoistic with the main goal of personal well-being (health, absence of punishment). In that sense, social distancing seems to be mainly driven by other-oriented psychological and social factors.

The COVID-19 pandemic was a threat to everyone’s health. Moreover, disregarding social distancing regulations may have led to fines. It is thus remarkable that self-oriented factors played a minor role for preventive behavior. With increasing fear of infection, people reported more compliance with social distancing. This finding supports a protective function of fear (Harper et al., 2020). Yet, the current study extends previous research by demonstrating that the effect of fear is subordinate to factors that concern other’s well-being. The second self-focused factor, fear of punishment, seems to be least effective in motivating people to keep social distance. This finding aligns with the observation that the German population moved less even before fines were implemented (Institute for Health Metrics and Evaluation, 2020). In addition, it suggests that future health-promoting projects could focus less on deterrence and more on affiliation.

Why are other-oriented factors most strongly related to social distancing during a pandemic? When faced with a threat, humans are inclined to seek contact (Dezecache et al., 2020). The pandemic can be regarded as a threat to one’s survival, which might activate the attachment system and thereby increase proximity seeking to caring others (Bowlby, 1969/1982; Mikulincer and Shaver, 2003). Because the attachment system particularly calls for proximity to supportive others, empathy for loved ones rather than empathy in general might be central for behavior. Being faced with the restriction of exactly what is needed during a crisis, namely social contact, might enforce this need of seeking contact even more. In addition, as COVID-19 was a threat to everyone, the sense of a “common fate” might have given rise to a motivation of collective action (Drury, 2018). Hence, the shared experience of a global threat and physical contact restrictions possibly amplified people’s social need and thereby attuned people to other’s well-being.

Beyond informing about social distancing, the current study refines theories of moral identity. Importantly, internalization of moral identity correlated positively with social distancing. This finding underlines the importance of moral identity for prosocial behavior even in a crisis. Concurrently, the effect of moral identity vanished when controlling for other factors. The idea that moral identity bridges the gap between moral judgment and behavior (Blasi, 1983) is thus not supported. Instead, with increasing moral identity, participants also increasingly reported social distancing to be morally relevant. It seems that the moral significance of the situation was very high, particularly for people with a strong moral identity. We will expand on this point below. In addition, moral identity correlated positively with all other-oriented factors. In line with the conceptual background of moral identity (Lapsley and Narvaez, 2004), this pattern indicates that with increasing moral identity, interpersonal aspects become more relevant.

Moral identity moderated the relation between moral judgment and social distancing, but the direction of the moderation was contrary to what we expected. For all levels of moral identity, moral judgment was positively related to social distancing, as hypothesized. But the relation was strongest for people with a low moral identity. For people with a low moral identity, judging certain behavior as morally relevant seems to be particularly important for behavior. Their moral judgment might act as a substitute for the low moral identity. With increasing moral identity, people might perceive more topics as morally relevant, as morality is a central aspect of their identity (Hardy and Carlo, 2011). Indeed, participants who reported a high moral identity also reported high moral judgments. This left little variance of moral judgment in people with a high moral identity that could explain social distancing. Overall, the moderating effect indicates that moral judgment and moral identity complement each other. Considering a behavior as moral can compensate for a low moral identity and still lead to moral behavior.

Our study shed light on the underlying factors that are associated with social distancing. Yet, to assess social distancing, we relied on participants’ self-report. Self-reports might have been biased by social desirability. Nevertheless, at least self-reports about behavioral compliance during the COVID-19 pandemic appear to be rather independent of social desirability bias (Larsen et al., 2020). Additionally, our sample was limited to people living in Germany. However, the COVID-19 pandemic is a worldwide phenomenon and countries react differently, leading to varying societal reactions. Investigating our question in a more diverse sample would be valuable. Moreover, internal consistency of our social distancing scale was rather low. As the items addressed different areas of social distancing (e.g. visiting elderly, meeting friends, distance in public space), this might suggest that the tendency to keep physical distance depends on the affected area. For example, the feasibility of keeping distance from elderly might differ from other areas due to potential family commitments. Nevertheless, we consider the overall social distancing score as an index of people’s general tendency to keep distance. It remains an open question whether some specific psychological or social factors are relevant for specific facets of social distancing.

Besides the theoretical interest, the current findings have practical relevance for handling a pandemic. While citizens adhered to the social distancing regulations rather reliably at the beginning, the acceptance declined (Betsch et al., 2020b). Hence, identifying the main factors that motivate people to follow the rules throughout a health-crisis is useful for politicians or healthcare professionals. Although our data are correlational and further support in experimental studies would be valuable, they highlight two factors. First, highlighting the moral significance of preventive behavior could be effective to promote it. Second, pointing towards the vulnerability of loved ones might increase adherence to restrictive regulations. These approaches could promote preventive behavior in times of a health crisis.

To conclude, this study underlines the social nature of humans, particularly the concern for close social relations, and the importance of moral considerations for everyday behavior. Even in face of a global pandemic, cognitive and affective other-oriented factors are more strongly related to preventive behavior than self-focused factors.

## Supplemental Material

sj-pdf-1-hpq-10.1177_1359105320980793 – Supplemental material for Prevention for oneself or others? Psychological and social factors that explain social distancing during the COVID-19 pandemic Click here for additional data file. Supplemental material, sj-pdf-1-hpq-10.1177_1359105320980793 for Prevention for oneself or others? Psychological and social factors that explain social distancing during the COVID-19 pandemic by Natalie Christner, Regina M Sticker, Lena Söldner, Maria Mammen and Markus Paulus in Journal of Health Psychology
