# Supplementary material for: Prevention for oneself or others? Psychological and social factors that explain social distancing during the COVID-19 pandemic
Source: J Health Psychol. 2020 Dec 10;27(6):1342–53. doi: 10.1177/1359105320980793 (PMC9036152; doi:10.1177/1359105320980793)
Supplement: sj-pdf-1-hpq-10.1177_1359105320980793 – Supplemental material for Prevention for oneself or others? Psychological and social factors that explain social distancing during the COVID-19 pandemic [file sj-pdf-1-hpq-10.1177_1359105320980793.pdf]

Supplemental Material

Prevention for oneself or others? Psychological factors that explain social distancing during the COVID-19 pandemic

Full list of items

Social Distancing

*: reverse coded; (D): Distractor items

**Original item**	**Translated from German**
Die COVID-19-Pandemie fordert Einschränkungen mancher Handlungen und bringt neue Regeln mit sich. Manche der Änderungen sind einfacher umzusetzen, manche schwieriger. Menschen gehen unterschiedlich mit den Beschränkungen und der Situation im Allgemeinen um. Wie ist es bei Ihnen?<br>Geben Sie bitte im Folgenden an, wie sehr die folgenden Aussagen für den Zeitraum der letzten zwei Wochen auf Sie zutreffen.	The COVID-19 pandemic demands restrictions of some behaviors and brings along new regulations. Some of the changes are easier and some are harder to implement. Individuals deal differently with the restrictions and the situation in general. How about you?<br>Please indicate in the following, how much the statements apply to you for the time period of the last two weeks.
1. Ich minimiere den physischen Kontakt zu anderen (sogenanntes "Social Distancing").	1. I minimize physical contact to others (so-called "Social Distancing").
2. (D) Ich informiere mich über die neusten medizinischen Entwicklungen in Bezug auf COVID-19 (z.B. Zahl der Infizierten, Impfstoff- und Medikamentenentwicklung).	2. (D) I inform myself about the latest medical developments related to COVID-19 (e.g. number of infections, vaccine and drug development).
3. (D) Ich nutze technische Möglichkeiten (Skype, WhatsApp, Telefon etc.), um mit meinen Freunden/meiner Familie in Kontakt zu bleiben.	3. (D) I use digital tools (Skype, WhatsApp, phone etc.) to stay in touch with my friends / family.
4.* Wenn es das Wetter zulässt, treffe ich mich draußen mit Freunden, die nicht in meinem Haushalt leben.	4.* In case of good weather, I meet outdoors with friends that do not live in my household.
5. (D) Ich nutze technische Möglichkeiten (Skype, Zoom, etc.) für meine Arbeit/Aufgaben.	5. (D) I use digital tools (Skype, Zoom, etc.) to do my work/tasks.
6.* Ich habe physischen Kontakt mit Familienmitgliedern, die nicht in meinem Haushalt leben.	6.* I am in physical contact with family members that do not live in my household.

7. (D) Es fällt mir leicht, meine Freizeit trotz der Ausgangsbeschränkungen nach meinen Vorstellungen zu gestalten.	7. (D) Despite the lockdown rules it is easy for me to organize my free time according to my own ideas.
8.* In meiner Freizeit besuche ich ältere Menschen (z.B. Eltern, Großeltern, ältere Freunde).	8.* I visit elderly people in my free time (e.g. parents, grandparents, older friends).
9. Im öffentlichen Raum (z.B. öffentlicher Nahverkehr, Supermarkt...) versuche ich, den empfohlenen Abstand von 1,5 Metern zu anderen einzuhalten.	9. I try to keep the recommended distance of 1.5 meters from others in public spaces (e.g. local public transport, supermarket ...).
10. (D) Ich kann unter den derzeitigen Bedingungen meiner Arbeit/meinen Aufgaben gut nachkommen.	10. (D) Under the current circumstances, I can complete my work/tasks without any problems.
11.* Ich treffe mich Zuhause mit Freunden.	11.* I meet up with friends at home.

Moral judgment

**Original item**	**Translated from German**
Zu den Maßnahmen im Umgang mit COVID-19 gehört auch das sog. "Social Distancing". Um die Weiterverbreitung des Virus zu verlangsamen, sind Menschen dazu aufgefordert, einen größeren Abstand zu Anderen einzuhalten und sich nicht mit Freunden und Familie zu treffen. Wie stehen Sie zu solchen Maßnahmen?<br>Wie sehr stimmen Sie den folgenden Aussagen zu?	“Social Distancing” is one of the methods for dealing with COVID-19. To slow down the spreading of the virus, people are asked to keep greater distance to others and to not meet friends and family. What do you think about such methods?<br>How much do you agree with the following statements?
1. "Social Distancing" ist moralisch richtig und gut.	1. "Social Distancing" is morally right and good.
2. Jemand, der sich nicht an das "Social Distancing"hält, handelt unmoralisch.	2. Someone who does not stick to "Social Distancing" acts immoral.
3. Auch wenn es keine staatlichen Verordnungen zu "Social Distancing"geben würde, wäre "Social Distancing"in der aktuellen Situation moralisch geboten.	3. Even if there were no state regulations about "Social Distancing", "Social Distancing" would be morally required in the current situation.
4. Es ist die moralische Pflicht einer bzw. eines jeden, "Social Distancing"einzuhalten.	4. It is the moral obligation of everyone to adhere to "Social Distancing".

Moral identity

Questionnaire by Aquino & Reed (2002)

Empathy (general)

Original item	Translated from German
Im folgenden Abschnitt interessieren wir uns für Ihre Gefühle im Bezug auf die momentane Situation.	In the following, we are interested in your feelings regarding the current situation.
1. Ich mache mir große Sorgen um diejenigen, die besonders durch COVID-19 gefährdet sind.	1. I am very concerned about those most vulnerable to COVID-19.
2. Ich habe großes Mitgefühl mit denjenigen, die besonders durch COVID-19 gefährdet sind.	2. I feel compassion for those most vulnerable to COVID-19.
3. Das, was mit denjenigen passieren kann, die besonders durch COVID-19 gefährdet sind, berührt mich sehr.	3. I am quite moved by what can happen to those most vulnerable to COVID-19.

Empathy (loved ones)

Original item	Translated from German
1. Ich mache mir große Sorgen um Familienangehörige oder Freunde, die besonders durch COVID-19 gefährdet sein könnten.	1. I am very concerned about family members or friends who are especially vulnerable to COVID-19.
2. Ich habe große Angst, dass mir nahestehende Personen mit COVID-19 infiziert werden könnten.	2. I am very scared that my loved ones could contract COVID-19.
3. Ich mache mir Sorgen, ob Familienangehörige oder Freunde auch genug tun, um sich vor einer Infektion mit COVID-19 zu schützen.	3. I worry whether family members or friends do enough to protect themselves from a COVID-19 infection.

Fear of infection

Original item	Translated from German
Die Menschen gehen unterschiedlich mit der Gefahr einer Infektion mit COVID-19 um.	People handle the danger of contracting COVID-19 differently. How about you?

Wie ist es bei Ihnen?<br>Wie sehr stimmen Sie folgenden Aussagen zu?	How much do you agree with the following statements?
1. Ich mache mir oft Sorgen, dass ich mich mit COVID-19 infizieren könnte.	1. I often worry that I might contract COVID-19.
2. Ich achte öfter darauf, ob ich die Symptome der COVID-19 Erkrankung haben könnte.	2. I often check if I might have symptoms of COVID-19.
3. Ich habe große Angst, dass ich mich mit COVID-19 infizieren könnte.	3. I am very scared that I might contract COVID-19.
4. Ich denke viel darüber nach, wie ich verhindern könnte, mit COVID-19 infiziert zu werden.	4. I think a lot about how I could prevent contracting COVID-19.

Fear of punishment

**Original item**	**Translated from German**
Die Einhaltung der Ausgangsbeschränkungen wird seit einigen Wochen von den Ordnungsämtern und der Polizei überwacht und durchgesetzt. Wer sich nicht an die Ausgangsbeschränkungen hält, begeht eine Ordnungswidrigkeit und muss mit Bußgeld rechnen. Wie wirkt sich das auf Sie aus?<br>Wie sehr stimmen Sie den folgenden Aussagen zu?	The order enforcement offices and the police surveil and enforce the lockdown rules since a few weeks ago. Those who do not comply with the restrictions commit minor offences and can be fined. How does this affect you?<br>How much do you agree with the following statements?
1. Weil es Bußgelder beim Verstoß gegen Ausgangsbeschränkungen und Mindestabstände gibt, achte ich mehr darauf, mich an das „Social Distancing“ zu halten.	1. Because there are fines when violating lockdown and distancing rules, I make sure to comply to "Social Distancing".
2. Ich habe Sorge, dass ich bestraft werde, wenn ich mich nicht an die staatlich verordneten Ausgangsbeschränkungen und das"Social Distancing"halte.	2. I worry that I might get fined if I do not adhere to the state-ordered lockdown rules and "Social Distancing".
